# Correction: Integrated biosensors for monitoring microphysiological systems

**DOI:** 10.1039/d4lc90026j

**Published:** 2024-03-19

**Authors:** Lei Mou, Kalpana Mandal, Marvin Magan Mecwan, Ana Lopez Hernandez, Surjendu Maity, Saurabh Sharma, Rondinelli Donizetti Herculano, Satoru Kawakita, Vadim Jucaud, Mehmet Remzi Dokmeci, Ali Khademhosseini

**Affiliations:** a Terasaki Institute for Biomedical Innovation 1018 Westwood Blvd Los Angeles California USA mdokmeci@terasaki.org khademh@terasaki.org; b Department of Clinical Laboratory, Third Affiliated Hospital of Guangzhou Medical University, Guangzhou Medical University No. 63 Duobao Road, Liwan District Guangzhou Guangdong P. R. China; c Department of Bioprocess and Biotechnology Engineering, School of Pharmaceutical Sciences, São Paulo State University (UNESP) Araraquara SP 14801-902 Brazil

## Abstract

Correction for ‘Integrated biosensors for monitoring microphysiological systems’ by Lei Mou *et al.*, *Lab Chip*, 2022, **22**, 3801–3816, https://doi.org/10.1039/D2LC00262K.

The authors provide additional information to clarify some of the statements made in the published article.

On page 2, the sentence “A biosensor is a device that utilizes a bioreceptor to “translate” biological events into quantifiable signals.^36^” should be revised as follows: “A biosensor is a device that employs a bioreceptor with selectivity for a specific target or analyte, translating this biorecognition event into a measurable signal.^36^ For the purpose of this review, we use the term “biosensors” to encompass a broader scope including not only biosensors that use a bioreceptor but also chemical and physical sensors as, within the microphysiological systems (MPS) community, sensors monitoring either the environment, cells, or stimuli response are often referred to as “Biosensors”.”

On page 2, “For voltammetric sensors, the change in electric current between a working and reference electrode as a function of applied potential is measured.” should be replaced with “For voltammetric sensors, the current flow between a working and a counter electrode as a function of applied potential is measured.”

On page 2, “Potentiometric sensors detect the concentration of analytes by monitoring the changes in potential between the reference and working electrodes while keeping a constant current.” should be replaced with “Potentiometric sensors detect the concentration of analytes by monitoring the changes in potential between the reference and working electrodes while keeping the current negligible (close to zero).”

On page 3, “Voltammetric/amperometric biosensors can monitor the binding activity across a range of applied potentials/currents by detecting well-defined current/potential peaks.” should be revised as follows: “Voltammetric biosensors operate by measuring current as a function of controlled time-dependent applied potential whereas amperometric biosensors measure current at a controlled applied potential.^1^”

Regarding the Electrochemical biosensors section on page 2 and page 3, our overall aim was to briefly describe the basics of biosensors used in the organ-on-a-chip field and discuss the integration of sensors to monitor MPS. Therefore, we kept the detailed description of “general biosensors” to a minimum and focused on reviewing biosensors integrated into MPS.

In Table 1, the following changes should be made: the enzyme “lactase” should be replaced with “lactate oxidase”; ref. 62 and 63 should be replaced with 2 and 3 listed below, respectively; “NM” should read “nM”; and “Probe” should be replaced with “Bioreceptor.”

In addition, “Limitation in targets” in the table referred to the limitation in pH target ranges, which usually do not measure the full range from 0 to 14. Overall, this column listed the advantages and limitations of each biosensor but did not compare them to avoid bias in interpretation by summarizing each work using the authors' words where appropriate. Lastly, the fast response attribute refers to the time needed from data acquisition to outcome measure reading, which varies between different biosensors.

On pages 7 and 12, the word “physical” should be replaced with “microenvironmental” for the following sentences: “Physical parameters (*i.e.*, oxygen, temperature, and pH) are widely monitored and studied using commercial and customized physical sensors.” (page 7); “Simultaneously, physical parameters (*i.e.*, pH, oxygen, and temperature) were monitored in their system.” (page 7); and “This is easy for physical parameters such as temperature, pH, and oxygen because they exhibit a fast sample-to-answer time.” (page 12).

On page 9, “For monitoring biochemical parameters, the saturation of the electrode sensor surface is a potential problem. This hinders the long-time stability of these sensors.” should be replaced with “For monitoring biochemical parameters, the saturation of the electrode sensor surface is a potential problem. This factor not only hinders the long-term reusability of these sensors but also limits their dynamic range.”

On page 12, “This is particularly important for the Clark electrode was utilized to measure oxygen.^148^” should be replaced with “This could be particularly important when using a Clark electrode for measuring oxygen.^148^”.

The Royal Society of Chemistry apologises for these errors and any consequent inconvenience to authors and readers.

## Supplementary Material
